# Radiologic Assessment of the Sinonasal Tract, Nasopharynx and Mastoid Cavity in Patients with SARS-Cov-2 Infection Presenting with Acute Neurological Symptoms

**DOI:** 10.1177/0003489421995070

**Published:** 2021-11

**Authors:** Gul Moonis, Ryan Mitchell, Betsy Szeto, Anil K. Lalwani

**Affiliations:** 1 Department of Radiology, Columbia University Irving Medical Center, New York, NY, USA; 2 Department of Otolaryngology—Head and Neck Surgery, Columbia University Vagelos College of Physicians and Surgeons, New York, NY, USA; 3 Department of Otolaryngology—Head and Neck Surgery, Division of Otology, Neurotology, & Skull Base Surgery, Columbia University Irving Medical Center, New York, NY, USA

**Keywords:** COVID-19, sinus infection, olfactory cleft, mastoid cavity

## Abstract

**Background:**

Acute neurological sequela in patients with COVID-19 infection include acute thromboembolic infarcts related to cytokine storm and post infectious immune activation resulting in a prothrombotic state. Radiologic imaging studies of the sinonasal tract and mastoid cavity in patients with COVID-19 infection are sparse and limited to case series. In this report, we investigate the radiologic involvement of nasal cavity, nasopharynx, paranasal sinuses, and mastoid cavity in patients with SARS-CoV-2 infection who presented with acute neurological symptoms.

**Methods:**

Retrospective review of medical records and neuroradiologic imaging in patients diagnosed with acute COVID-19 infection who presented with acute neurological symptoms to assess radiologic prevalence of sinus and mastoid disease and its correlation to upper respiratory tract symptoms.

**Results:**

Of the 55 patients, 23 (42%) had partial sinus opacification, with no evidence for complete sinus opacification. The ethmoid sinus was the most commonly affected (16/55 or 29%). An air fluid level was noted in 6/55 (11%) patients, most commonly in the maxillary sinus. Olfactory recess and mastoid opacification were uncommon. There was no evidence of bony destruction in any of the studies, Cough, nasal congestion, rhinorrhea, and sore throat were not significantly associated with any radiological findings.

**Conclusion:**

In patients who present with acute neurological symptoms, COVID-19 infection is characterized by limited and mild mucosal disease within the sinuses, nasopharynx and mastoid cavity.

**Level of Evidence:**

4

## Introduction

Human coronavirus (HCoV), a family of single positive stranded RNA viruses, including 229E, OC43, NL63, HKU1, SARS, MERS (Middle East respiratory syndrome) and SARS-CoV-2 (COVID-19, coronavirus disease 2019) are a frequent cause of the common cold, respiratory infection, and middle ear pressure abnormalities.^1-3^ In one study of children with HCoV, the most common findings were cough, rhinorrhea, tachypnea, fever, abnormal breath sounds, and hypoxia.^4^ URI symptoms are also common among patients with COVID-19 infection, including cough, sore throat, congestion or runny nose.^5^ Other symptoms include fever or chills, shortness of breath, fatigue, muscle or body aches, headache, olfactory and gustatory dysfunction, muscle aches, nausea, vomiting, and diarrhea.^6^ Thromboembolic infarctions related to hypercoagulability and cytokine storm are also well described in COVID-19 patients and may be the initial presentation of the infection.^7,8^

Presence of upper respiratory symptoms is consistent with the presence of virus particles in the nasal cavity and nasopharynx.^9^ The virus is transmitted between individuals through respiratory droplets produced by the infected person, mild sneezing, coughing, or talking.^10^ Based on single-cell RNA analysis, the nasal cavity has been shown to have among the highest level of expression of ACE2, the host cell surface receptor for SARS-SoV-2, within the aerodigestive tract.^11^ Nasal shedding of live virus is usually quite high early in the course of COVID-19, precedes lower respiratory tract viral shedding, and may even continue after the virus is no longer detected from the lower respiratory tract.^12^ Increased viral load in the nasopharynx and oropharynx is the basis for detection of SARS-CoV-2 by real-time reverse-transcriptase polymerase-chain-reaction (RT-PCR) assays collected by nasal and oropharyngeal swabs.^9^

The aim of our study was to determine the prevalence of upper respiratory tract symptoms and radiologic findings pertaining to the upper respiratory tract (paranasal sinuses, mastoid cavity and nasopharyngeal lymphoid hypertrophy) in COVID-19 patients who presented with acute neurological symptoms.

## Material and Methods

The study was approved by the institutional review board, which granted waiver of informed consent. We identified 100 consecutive COVID-19 patients who underwent neuroimaging examinations (computerized tomography (CT) or magnetic resonance imaging (MRI)) between March 25, 2020 and April 24, 2020. All of these patients were confirmed as COVID-19 positive on RT-PCR assay of nasopharyngeal or oropharyngeal swab specimens. Of the 100 consecutive patients, 38 were excluded from the study due to presence of nasogastric tube and/or endotracheal tube, 4 were excluded because repeat RT-PCR tests obtained closer to the time of the imaging study were either negative or indeterminate, 2 were excluded because their RT-PCR test was obtained greater than 10 days prior to the imaging study, and 1 was excluded due to prior paranasal sinus surgery. Of the 55 patients who underwent neuroimaging, 54 had CT and 1 had MRI. The 54 CT scans included 50 CT heads, 1 maxillofacial CT, 1 sinus CT, 1 orbit CT, and 1 CT neck. All exams were performed using standard MDCT protocol. The maxillary sinuses were excluded from the field of view on 6 patients, the olfactory recess was excluded from the field of view on 1 patient, and the nasopharynx was excluded from the field of view on 1 patient. The portion of the radiologic review that could not be performed was not included in the data set and the total patient number for that specific category was adjusted accordingly. In the remaining exams, the areas of interest (paranasal sinuses, mastoid air cells, nasopharynx and olfactory recess were adequately visualized.

Of these 55 patients, 29 (53%) were male and 26 (47%) were female. The age of the patients ranged from 24 to 92 with a mean of 68 years. All, but one, of the patients were admitted to the hospital.

Although the neuroimaging in this cohort was performed for acute neurological findings, we reviewed the patient charts for additional symptoms. Neurologic symptoms included, but were not limited to, altered mental status, somnolence, fatigue, and neurologic deficits. Symptoms in 3 additional categories were assessed: respiratory system, fever and gastrointestinal system. Respiratory symptoms included, but were not limited to, shortness of breath, hypoxia, sore throat, cough, rhinorrhea, nasal congestion, and new onset loss of smell or altered sense of taste. Fever could have been self-reported, documented by outside medical facilities including a nursing home or urgent care center, or noted upon admission. Gastrointestinal symptoms included abdominal pain, bloating, nausea, vomiting, or diarrhea. We specifically focused our review on the upper respiratory symptoms, including cough, sore throat, nasal congestion, rhinorrhea, and new onset loss of smell or altered sense of taste. We also reviewed the chart for history of chronic sinus disease or ear infections.

**Figure 1.**
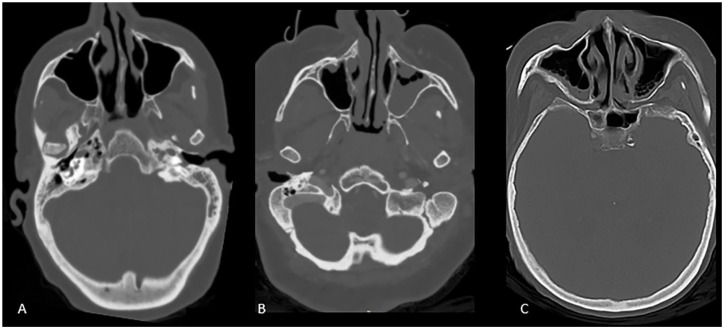
Sinus opacification grades. Axial CT scan images demonstrate normally aerated sinus (A), partial bilateral left greater than right maxillary sinus opacification (B) and partial bilateral maxillary sinus opacification with fluid level on the right (C).

**Figure 2.**
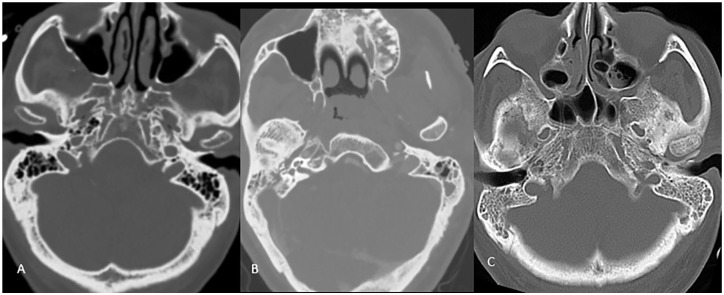
Mastoid opacification grades. Axial CT scan images demonstrate normally aerated, partially opacified left greater than right mastoid air cells and bilateral completely opacified mastoid air cells in images A, B, and C respectively.

**Figure 3.**
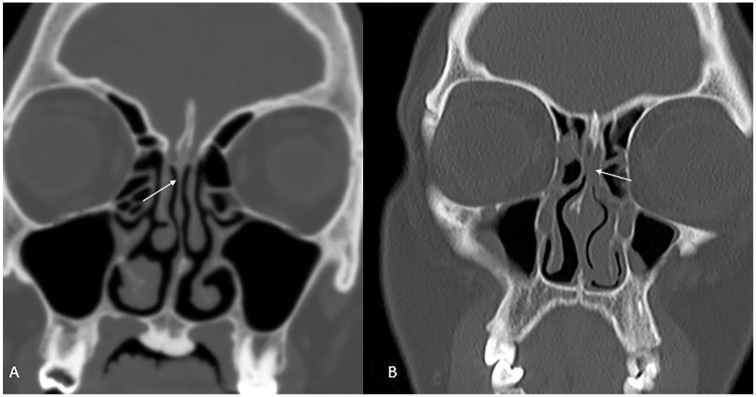
Olfactory recess grades. Coronal CT scan images demonstrates an aerated olfactory recess in (A) and bilaterally opacified olfactory recess in (B) (white arrows).

**Figure 4.**
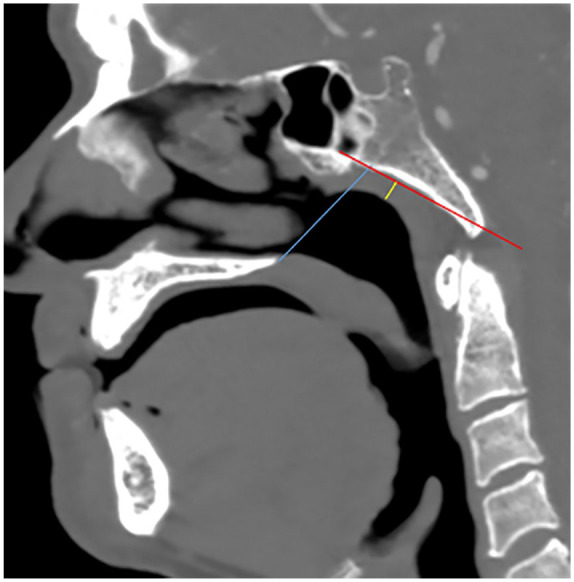
Nasopharyngeal thickness using the adenoidal/nasopharyngeal ratio (Fujioka method). Adenoid measurement was made by drawing a perpendicular line from a line drawn along the straight part of the anterior margin of basiocciput (red line) to a point of maximal convexity of adenoid (yellow line). Nasopharyngeal measurement (blue line) was determined by drawing a line from the anterior inferior edge of sphenobasioccipital synchondrosis to the posterior superior margin of the hard palate. A/N Ratio was then determined by dividing adenoidal measurement with nasopharyngeal measurement.

All images were reviewed in consensus by a board-certified neuroradiologist with 19 years experience and a senior radiology resident for the extent and severity of sinus disease, olfactory recess opacification, nasopharyngeal thickness, and mastoid air cell opacification. The maxillary, ethmoid, frontal, and sphenoid were evaluated individually. The Lund Mackay scoring scheme for the extent of sinus disease was used for the paranasal sinuses and mastoid cavity; LM score is a 3-point scoring system used to describe sinus opacification (none = 0; partial opacification = 1; complete opacification = 2) (Figures 1 and 2). Within the sinuses and mastoid cavity, the presence or absence of air fluid level and bony destruction were noted. Olfactory recess opacification was noted as absent (0) or present (Figure 3). Nasopharyngeal thickness was assessed using a modification of the adenoidal nasopharyngeal (A/N) ratio as proposed by Fujioka et al^13^ (Figure 4). The measurements were made on the lateral scout topogram of the CT scan of the brain in 47 patients, sagittal midline reconstruction from CT Angiogram of the circle of Willis in 5 patients, sagittal midline image of a maxillo-facial CT scan in 1 patient and sagittal midline T1 weighted MRI image on 2 patients. In the age group of our patient cohort, adenoidal tissue was expected to have regressed and a ratio above 0.20 was classified as abnormal.^14^ We further graded the severity of the nasopharyngeal thickness as follows: a ratio of >0.20 to 0.40 as mild thickness, >0.40 to 0.60 as moderate thickness and >0.60 as severe thickness.

*Statistical analysis*: The imaging findings of the patients with the symptom of interest were compared to those without. For categorical variables, the data are presented as frequency and percent. The Chi-Square test was used when all expected cell counts were 5 or greater. When expected cell counts were less than 5, Fisher’s exact test was used. For patients with sinus disease, the median and interquartile range of the number of sinuses involved are presented, and the Mann–Whitney *U* test was used to compare those with the symptom of interest to those without. For all analyses, *P* < .05 was considered statistically significant. All analyses were conducted using SAS 9.4 (SAS Institute, Cary, NC).

## Results

Among the 55 patients, RT-PCR was positive within the range from 9 days before and up to 3 days after the imaging study, with 96% (53/55) being positive within ±3 days of the neuroimaging study. All patients presented with acute neurological symptoms. Fourteen patients only had neurological symptoms. Three patients had neurological and gastrointestinal symptoms. Eleven patients had neurological and respiratory symptoms. Two patients had neurological symptoms and fever. Twenty-five patients had neurological symptoms and a combination of fever, gastrointestinal or respiratory symptoms. The most common combination was neurological symptoms, respiratory symptoms and fever. None of the patients presented with new onset of loss of smell or altered sense of taste. None of the patients in our cohort had history of sinus disease or ear infections.

Of the 55 patients, 23 (41.8%) had sinus disease; it was mild in 20 patients and moderate to severe in 3 patients (Table 1). Among the patients with sinus disease, the majority had single sinus disease: single sinus was involved in 13 or 57%; 2 sinuses in 2 or 9%, 3 sinuses in 5 or 22%, and 4 sinuses in 3 or 13% of patients. The ethmoid sinus was the most commonly affected (16/55 or 29%), followed by maxillary sinus (11/49 or 22%) and sphenoid sinus (10/55 or 18%). Frontal sinus was the least affected (7/55 or 13%). An air fluid level was noted in 6/55 (11%) patients, most commonly in the maxillary sinus (Figure 1). One patient had air fluid level in all 4 sinuses. Olfactory recess opacification was observed in 4/54 patients (Figure 3). There was no evidence of bony destruction in any of the studies.

**Table 1.** Extent of Sinus Disease, Mastoid Opacification, Olfactory Recess Opacification, and Nasopharyngeal Thickening.

Anatomic area	No opacification (%)	Partial opacification (%)	Complete opacification (%)	Air fluid level (%)	Bone destruction (%)	Total
Any sinus	32 (58)	23 (42)	0	6 (11)	0	55
Maxillary	41 (83)	8 (17)	0	3 (6)	0 (0)	49^†^
Ethmoid	39 (70)	16 (30)	0	2 (4)	0 (0)	55
Frontal	48 (87)	7 (13)	0	2 (4)	0 (0)	55
Sphenoid	45 (82)	10 (18)	0	2 (4)	0 (0)	55
Mastoid cavity	51 (93)	3 (6)	1 (2)	0 (0)	0 (0)	55
Olfactory recess	50 (93)		4 (7)			54^†^
	Normal (%) (A/N<0.20)	Mild thickening (%) (0.20<A/N≤0.40)	Moderate thickening (%) (0.40<A/N≤0.60)	Severe thickening (%) (A/N>0.60)	Total	
Nasopharyngeal thickness	25 (46.3)	22 (40.7)	5 (9.3)	2 (3.7)	54^†^	

Abbreviations: A/N, adenoidal nasopharyngeal ratio.

† Due to missing data, for Maxillary Sinus, N = 49, for Olfactory Recess, N = 54, and Nasopharyngeal Thickness, N = 54.

Mastoid opacification was noted in 4/55 (7%) patients, mild in 3 (5%) and moderate to severe in 1 (2%) (Figure 2).

The nasopharynx was normal in size in 25 (46%), mildly thickened in 22 (41%), moderate thickened in 5 (9%) and severely thickened in 2 (4%) patients (Figure 4).

### Correlation of Symptoms with Imaging Findings

*Nasal Congestion*: One patient had nasal congestion mentioned as a symptom; this patient demonstrated mild sinus disease in all but the frontal sinuses (Table 2). There was no statistically significant difference in radiologic findings between patients with and without nasal congestion.

**Table 2.** Imaging Findings in Patients with and without Nasal Congestion.

	Nasal congestion present (N = 1)	Nasal congestion absent (N = 54)	Total (N = 55)†	*P*-value*
Sinus disease, N (%)	1 (100.0)	22 (40.7)	23 (41.8)	.42
Number of sinuses involved, Median (IQR)	3 (3-3)	1 (1-3)	1 (1-3)	.36
NP thickening, N (%)	1 (100.0)	28 (52.8)	29 (53.7)	1.00
Olfactory recess opacification, N (%)	1 (100.0)	3 (5.7)	4 (7.4)	.07
Mastoid opacification, N (%)	0 (0)	4 (7.4)	4 (7.3)	1.00

Abbreviations: IQR, interquartile range; NP, nasopharyngeal.

† Due to missing data, for NP thickening, N = 54, and for olfactory recess opacification, N = 54.

* For categorical variables, Fisher’s Exact test was used. When median (interquartile range) is presented, Mann–Whitney *U* test is used.

*Rhinorrhea*: Two patients had rhinorrhea as one of their initial symptoms (Table 3). Neither of these patients demonstrated any imaging findings. There was no statistically significant difference in radiologic findings between patients with and without rhinorrhea.

**Table 3.** Imaging Findings in Patients with and without Rhinorrhea.

	Rhinorrhea present (N = 2)	Rhinorrhea absent (N = 53)	Total (N = 55)†	*P*-value*
Sinus disease, N (%)	0 (0)	23 (43.4)	23 (41.8)	.50
Number of sinuses involved, median (IQR)	-	1 (1-3)	1 (1-3)	-
NP thickening, N (%)	2 (100.0)	27 (51.9)	29 (53.7)	.49
Olfactory recess opacification, N (%)	0 (0)	4 (7.7)	4 (7.4)	1.00
Mastoid opacification, N (%)	0 (0)	4 (7.5)	4 (7.3)	1.00

Abbreviations: IQR, interquartile range; NP, nasopharyngeal.

† Due to missing data, for NP thickening, N = 54, and for olfactory recess opacification, N = 54.

* For categorical variables, Fisher’s Exact test was used. When median (interquartile range) is presented, Mann–Whitney *U* test is used.

*Sore Throat*: Two patients had complained of sore throat (Table 4). Neither of these patients demonstrated any imaging abnormalities. There was no statistically significant difference in radiologic findings between patients with and without sore throat.

**Table 4.** Imaging Findings in Patients with and without Sore Throat.

	Sore throat present (N = 2)	Sore throat absent (N = 53)	Total (N = 55)†	*P*-value*
Sinus disease, N (%)	0 (0)	23 (43.4)	23 (41.8)	.50
Number of sinuses involved, median (IQR)	-	1 (1-3)	1 (1-3)	-
NP thickening, N (%)	0 (0)	29 (54.7)	29 (53.7)	.46
Olfactory recess opacification, N (%)	0 (0)	4 (7.7)	4 (7.4)	1.00
Mastoid opacification, N (%)	0 (0)	4 (7.5)	4 (7.3)	1.00

Abbreviations: IQR, interquartile range; NP, nasopharyngeal.

† Due to missing data, for NP thickening, N = 54, and for olfactory recess opacification, N = 54.

* For categorical variables, Fisher’s Exact test was used. When median (interquartile range) is presented, Mann–Whitney *U* test is used.

*Cough*: 28 patients had cough as a symptom at presentation (Table 5). Among the 28, 10 or 36% had sinus disease while 18 or 64% had no evidence of sinus disease. The patient with the most advanced sinus disease presented with symptoms that included cough. Among patients with and without cough, there was no significant differences in the presence of sinus disease, nasopharyngeal thickness, olfactory recess opacification, or mastoid cavity disease.

**Table 5.** Imaging Findings in Patients with and without Cough.

	Cough present (N = 28)	Cough absent (N = 27)	Total (N = 55)†	*P*-value*
Sinus disease, N (%)	10 (35.7)	13 (48.1)	23 (41.8)	.35
Number of sinuses involved, median (IQR)	1.5 (1-3)	1 (1-3)	1 (1-3)	.73
NP thickening, N (%)	16 (57.1)	13 (50.0)	29 (53.7)	.60
Olfactory recess opacification, N (%)	2 (7.4)	2 (7.4)	4 (7.4)	1.00
Mastoid opacification, N (%)	2 (7.1)	2 (7.4)	4 (7.3)	1.00

Abbreviations: IQR, interquartile range; NP, nasopharyngeal.

† Due to missing data, for NP thickening, N = 54, and for olfactory recess opacification, N = 54.

* For categorical variables, Fisher’s Exact test is used when expected cell counts <5, otherwise, the Chi-Square test is used. When median (interquartile range) is presented, the Mann–Whitney *U* test is used.

## Discussion

In this large radiologic study of patients admitted with acute neurological symptoms related to COVID-19 infection (but not presenting with upper or lower respiratory symptoms), we show that despite the presence of high viral load within the nasal cavity and nasopharynx, there is limited evidence of disease within the sinuses, nasopharynx or mastoid cavity. Significant nasopharyngeal thickness or olfactory cleft opacification was not seen in the majority of our cases. The mean age of our patient cohort was 68 years, well beyond the age group by which the normal adenoid tissues should regress, so the incidence of moderate to severe nasopharyngeal thickening in our study was likely not physiologic or age related. Limited sinus inflammatory disease, in most cases restricted to a single sinus, was observed in about 40% of patients; the most common sinus involvement was the ethmoid sinus followed by maxillary and sphenoid sinus. Air fluid level suggestive of acute sinusitis was present in only 11% of our cases. These findings of limited mucosal disease in COVID-19 patients is different from the typical findings of thickening of the walls of the nasal passages, engorged turbinates and sinus infection seen with viral URI.^15^ These findings suggest that the COVID infection may be restricted to the epithelium of the upper respiratory tract without leading to obstruction of sinus osteomeatal complex or the Eustachian tube. Similar to our findings, there was absence of sinusitis in a small cohort of 6 patients with olfactory dysfunction; and, only 1/3 had evidence of olfactory cleft opacification.^16^

Clinically, COVID-19 patients in this series had limited upper respiratory tract symptoms; cough—a symptom of both upper and lower airway disease, was the most common complaint. On the other hand, nasal congestion, rhinorrhea and sore throat were infrequent (<10%). These findings are consistent with many COVID-19 case series that have reported a paucity of sinonasal or URI symptoms, in contrast to lower respiratory tract and constitutional symptoms such as fever, cough, fatigue, shortness of breath, and myalgia.^17-21^ A systematic review of clinical presentation of COVID-19, focusing on upper airway symptoms revealed that pharyngodynia was present in 12.4% of patients, nasal congestion in 3.7%, and rhinorrhea was rare.^22^ Rhinorrhea has been reported in a small minority of patients in other case series of patients with COVID-19.^23-25^

Despite the high prevalence of neurological symptoms in our series, olfactory dysfunction was absent. This may be related to the acute neurological presentation in our cohort which may have made it difficult to obtain this history. While olfactory and gustatory dysfunction is an early symptom^6,26^ and in some patients anosmia may be the sole and presenting symptom of COVID-19,^27^ difference in prevalence has been reported. Much higher rates of olfactory dysfunction have been reported among the Europeans (34%-79%) compared to East Asians (4%-5%); these may be due to differences in ACE2 receptor expression within the nasopharynx.^28^ Imaging studies in patients with COVID-19 related anosmia have demonstrated findings ranging from olfactory cleft opacification, loss of volume in the olfactory bulb, and increased T2 FLAIR hyperintensity of the olfactory bulb and tracts.^21,29-32^ In our series of patients with acute neurological symptoms imaged predominantly by CT, opacification of the olfactory cleft was observed in only 7% of patients and it did not correlate with nasal symptoms or anosmia. This raises the possibility that COVID-19 related anosmia is likely not related to central nervous system pathology but may be secondary to local pathology in the sinonasal tract. However, none of our patients had a dedicated MRI of the olfactory bulb to assess for possible changes in signal or morphology, which is a limitation of our study. Another limitation of our study is that the patient’s could have had pre-existing sinus or mastoid opacification unrelated to COVID-19, although it was not noted in the chart.

## Conclusion

In patients presenting with neurological symptoms, COVID-19 infection is characterized by limited and mild mucosal disease within the sinuses, nasopharynx or mastoid cavity; acute infections and bone destruction are uncommon.
